# Hydatid detection using the near-infrared transmission angular spectra of porous silicon microcavity biosensors

**DOI:** 10.1038/srep44798

**Published:** 2017-03-20

**Authors:** Peng Li, Zhenhong Jia, Guodong Lü

**Affiliations:** 1 School of Physical Science and Technology, Xinjiang University, Urumqi 830046, China; 2 College of Information Science and Engineering, Xinjiang University, Urumqi 830046, China; 3 The First Affiliated Hospital of Xinjiang Medical University, Urumqi 830054, China

## Abstract

Hydatid, which is a parasitic disease, occurs today in many regions worldwide. Because it can present a serious threat to people’s health, finding a fast, convenient, and economical means of detection is important. This paper proposes a label- and spectrophotometer-free apparatus that uses optical biological detection based on porous silicon microcavities. In this approach, the refractive index change induced by the biological reactions of a sample in a porous silicon microcavity is detected by measuring the change in the incidence angle corresponding to the maximum transmitted intensity of a near-infrared probe laser. This paper reports that the proposed method can achieve the label-free detection of 43 kDa molecular weight hydatid disease antigens with high sensitivity.

Hydatid disease is a serious parasitic disease caused by the larvae of *Echinococcus granulosus* and *Echinococcus multilocularis* which affect both humans and animals^1^,^2^,^3^. The disease is most common in regions where animal husbandry is widespread. Parts of China, for instance, have some of the world’s highest incidences of hydatid disease. Cystic hydatid disease is distributed mainly over China’s northwest pastoral and farming regions. The infection rates of hydatid disease in a surveyed population reached 2.7% in Xinjiang^4^, where the number of infected cases of hydatid disease each year is 54 times the national average. In such areas, convenient and low-cost detection methods are particularly important for achieving early diagnosis. Currently, the most common methods of detection involve imaging with B-mode ultrasound or computerized tomography and immunological testing with indirect haemagglutination assays and enzyme-linked immunosorbent assay kits. Obtaining high-definition images of hydatid cysts requires sophisticated and expensive equipment. Because doctors who use this method need both access to high-definition images and extensive experience in reading and interpreting the images, the method is not feasible in remote and less-developed areas. The immunological detection method offers low costs; however, the results obtained when testing the same antibody samples can vary with different detection kits. Furthermore, the detection sensitivity is low, which makes it difficult to screen out patients with weak immune responses. Thus, it is particularly important to look for a high-sensitivity, low-cost, and convenient immunological detection method for hydatid disease.

Porous silicon is a favourable material because of its large specific surface area, good biological activity, and adsorption properties^5^,^6^. It can also be produced easily in a variety of photonic devices. Biosensors based on porous silicon are used in biomedical diagnostics, environmental monitoring, and veterinary and food-quality control^7^,^8^,^9^,^10^,^11^,^12^,^13^,^14^,^15^. Among porous silicon biosensors with different optical structures, the porous silicon microcavity (PSM) offers very high sensitivity^16^,^17^. The most commonly used method for the detection of biological materials using porous silicon sensors is the reflection spectrum method that using a spectrophotometer, detecting the resonance peak shift of the wavelength corresponding to the resonance peak of the reflection spectrum before and after the biological reaction in the PSM. This approach is based on the reaction of biomolecules in the PSM causing the refractive index of the device to increase, resulting in a redshift of the reflection spectrum. This method has also been applied to the detection of protein kinase P38 for diagnosing hydatidosis hydatid disease^18^.

However, for high sensitivity detection, a high resolution spectrophotometer is needed. For example, to measure a refractive index change of 10^−4^, the resolution of the spectrophotometer must reach 0.01 nm^19^. It is difficult to apply this method in an ordinary laboratory because of the expense and bulk of the spectrophotometer. To solve this problem, Li *et al*. proposed a spectrophotometer-free detection method based on the reflection angular spectrum to achieve highly sensitive detection for eight base pairs of DNA^19^. A refractive index change of 10^−4^ in PSM can be detected by the reflection angular spectrum method. The detection system includes only one He-Ne laser, one detector and one ordinary goniometer (with a resolution of 1′). However, there are still some problems to be solved in this method: porous silicon shows strong absorption to visible light, which has a great influence on the optical properties of PSM. Furthermore, the visible wavelength laser beam may be harmful to some biological samples.

In this paper, we propose a new optical biosensor label-free detection method, which has successfully achieved the immune detection of hydatid disease. The refractive index change induced by biological reactions in PSM is measured based on the transmission angular spectrum of near-infrared laser light. Because the absorption of the near-infrared laser in porous silicon is very small, the desirable optical characteristics of PSM devices are exhibited, and the detection sensitivity increases. As near-infrared light is located in the optically transparent window of the biological sample, no damage to the biological samples results^20^. The transmission angular spectrum method is easier than the reflection angular spectrum method. In the reflection angular spectrum method, to prevent the detector form blocking the incident light, there must be a certain distance between the sample and the detector. The orientation and position of the detector must be continuously adjusted to ensure that all of the reflected light can be received, which is inconvenient for sensor detection. In the transmission angular spectrum method, the sensor is fixed at the centre of the rotary stage of the goniometer, and the detector is stationary, so the transmitted light angle of the sensor can be measured easily by rotating the stage. In our work, 43 kDa cystic hydatid disease antigens have been detected by the near-infrared transmitted angular spectrum method based on PSM, where detection limit was 0.16 ng/ml.

## Material and Methods

### Preparation of PSMs and functionalization

PSMs were prepared by electrochemical anodization of highly doped p-type silicon (boron doped, 0.03–0.06 Ω·cm resistivity) in a mixed solution, 1:1 by volume, composed of 40% aqueous hydrofluoric acid (Tianjin Zhiyuan Chemical Reagent Co., Ltd., Tianjin, China) and 99% alcohol (Tianjin Fuyu Fine Chemical Co., Ltd., Tianjin, China).

**Figure 1:**
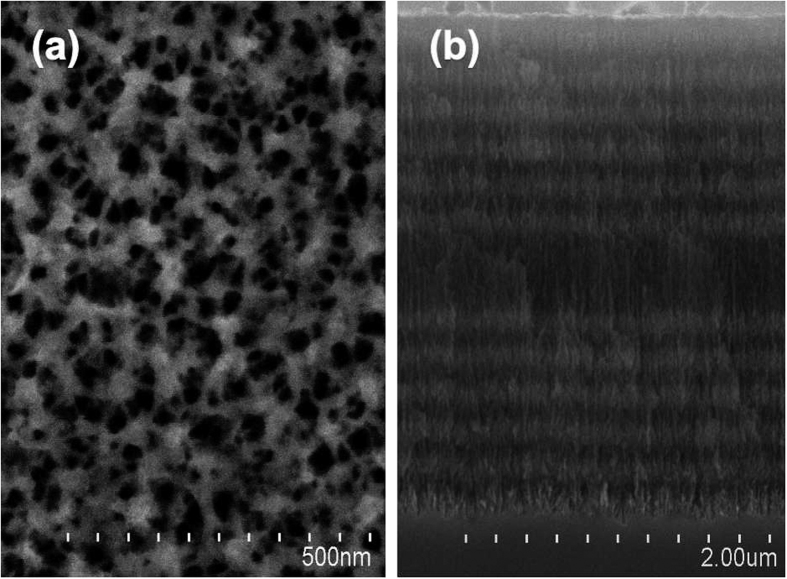
SEM image of (**a**) the top view of the surface (**b**) cross section of the PSM.

Silicon wafers (Tianjin Semiconductor Technology Research Institute, Tianjin, China) were cleaned with acetone (Tianjin Zhiyuan Chemical Reagent Co., Ltd., Tianjin, China), ethanol (Tianjin Zhiyuan Chemical Reagent Co., Ltd, Tianjin, China), and deionized (DI) water before use in the experiment. The silicon wafers were then anodized using the Labview program, which offered computerized control of the alternating current density and corrosion time. The current densities were 110 mA/cm^2^ and 60 mA/cm^2^; corrosion times were 2.5 s and 3.0 s. For the microcavity, the current density and corrosion time were 110 mA/cm^2^ and 5.0 s, respectively. Supplements increasing fluoride concentration and 3.0 s pauses were introduced after the formation of each layer to ensure that the corrosion was relatively uniform. Surface characterization data can be provided by SEM. Figure 1 shows that the diameter of the pores varies from 30 to 50 nm, and that the thickness of the PSM is approximately 3.5 μm.

After preparing the PSMs, the functional processing was necessary. The PSMs were put into a 40% hydrogen peroxide (Tianjin Zhiyuan Chemical Reagent Co., Ltd.) solution at 60 °C and placed in a vacuum drying oven (DZF-6050, Shanghai Yiheng Scientific Instrument Co., Ltd., Shanghai, China) for 3 h. Then, the PSMs were removed and repeatedly rinsed with DI water before being allowed to air dry. Next, the PSMs were soaked for 1 h in a 5% aminopropyltriethoxysilane solution (10:10:1 = DI water:methanol:APTES (99%, Sigma-Aldrich, St. Louis, MO, USA)), flushed repeatedly with DI water, and then baked at 100 °C for 10 min in a vacuum drying oven. Finally, the PSMs were put into a 2.5% solution of glutaraldehyde (19:1 = DI water:GA (50%, Aladdin Reagent Co., Ltd., Shanghai, China)) for 1 h at room temperature 22 °C and washed three times with phosphate buffer solution (PBS, Shanghai Sangon Biotech Co., Ltd., Shanghai, China) to remove excess glutaraldehyde. The PSMs were then washed again with DI water.

### Preparation of the biological probe and detection

After functionalization, hydatid antibody was selected as the probe. The PSMs were dropped into 20 μL of hydatid antibody (1:1000, obtained from rabbit serum, Xinjiang Key Laboratory of Hydatid Disease, Xinjiang, China) and then incubated for 2 h in a thermostat (Shanghai Jinghong Laboratory Instrument Co., Ltd., Shanghai, China), followed by cleaning with PBS and DI water. Finally, the PSMs were dipped into a concentration of 4% bovine serum albumin (BSA, Xinjiang Key Laboratory of Hydatid Disease, Xinjiang, China) for 1 h at 37 °C in the thermostat. The PSMs were then flushed with PBS and DI water and dried in air. The PSMs were placed on a goniometer stage with a measuring incidence angle *θ*_1_ corresponding to the maximum transmitted intensity of a near-infrared probe laser.

The PSMs were dropped separately into 20 μL of a solution containing different concentrations of hydatid antigen (Xinjiang Key Laboratory of Hydatid Disease, Xinjiang, China) and incubated in the thermostat for 1 h. Then, all samples were rinsed repeatedly with PBS and DI water and allowed to air dry. The PSMs were again placed on the goniometer stage with a measuring incidence angle *θ*_2_ corresponding to the maximum transmitted intensity of a near-infrared probe laser. The change in refractive index Δ*n* can be determined from the change of the laser incident angle: Δ*θ* = *θ*_2_ − *θ*_1_.

### Detection apparatus

**Figure 2:**
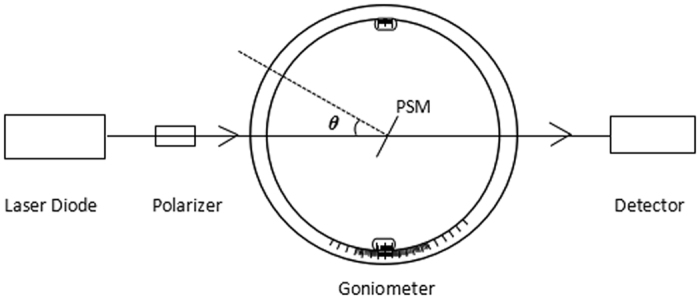
Experimental apparatus.

Figure 2 shows the experimental device used to implement the proposed optical transmission biological detection method based on the PSM. The device included a laser diode (Changchun New Industries Optoelectronics Tech Co., Ltd., Changchun, China), polarizer (Changchun No.1 Optical Instrument Factory, Changchun, China), goniometer (Changchun No.1 Optical Instrument Factory, Changchun, China), and detector (Thorlabs Inc., Newton, NJ, USA). The laser was a high-stability and power-tunable near-infrared semiconductor laser with a divergence angle of 1 mrad and a wavelength of 1550 nm.

### Theory and method

**Figure 3:**
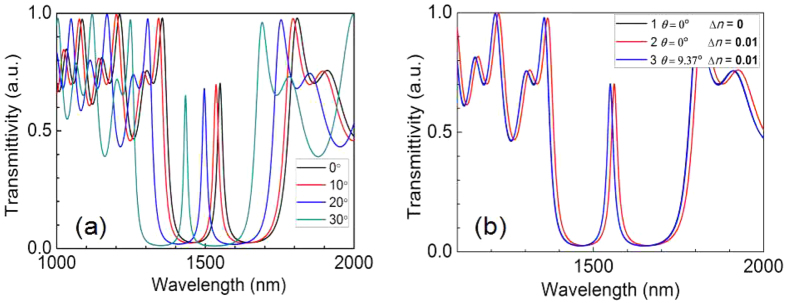
Transmission spectra for various angles. (**a**) PSM transmission spectra of for various incident angles. (**b**) Curve 1 represents the PSM transmission spectrum of vertical incidence (*θ* = 0°); curve 2 represents the occurrence in the PSM device of a biological reaction that caused the refractive index to increase to Δ*n* = 0.01, identical to a PSM transmission spectrum with vertical incidence (*θ* = 0°); curve 3 represents the PSM transmission spectrum after a certain rotary angle of incident light.

When the laser was incident on the PSM at different angles, the position of the transmission spectrum changed. In the experiment, the incident light was a transverse electric wave, and the refractive indices of the PS dielectric-layer were 1.21 and 1.52. The microcavity refractive index layer was also designed to exhibit a refractive index of 1.21. The optical thickness *nd* (where *n* is the refractive index, and *d* is the physical thickness) was set to two times *nd* of the dielectric layer PS. On both sides of the Bragg layer, the number of cycles was six. The reference wavelength *λ*_C_ was 1550 nm. The PSM device was designed to match these parameters. The incident light angle *θ* was adjusted to 0°, 10°, 20°, and 30° for theoretical simulation by Bruggeman effective medium approximation^21^ and the transfer matrix method^22^,^23^. The calculation results indicated that the transmission peaks were located at 1550 nm, 1536 nm, 1497 nm, and 1434 nm, respectively. Figure 3(a) shows the PSM transmission spectra, which demonstrate that when the incident angle increased, the transmission spectrum of the PSM underwent a blueshift.

**Figure 4:**
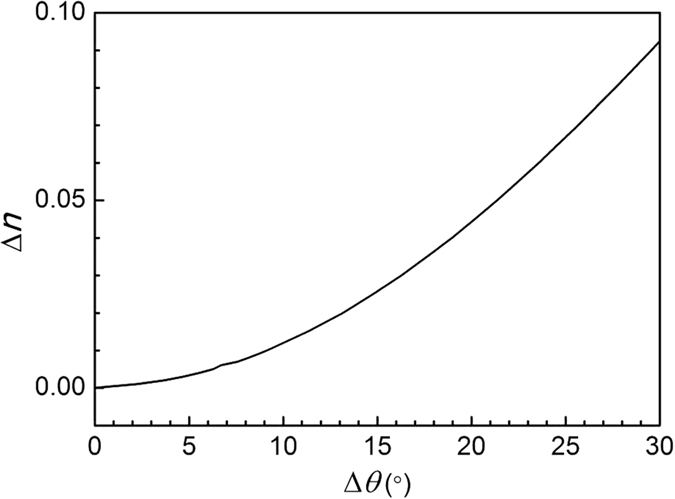
Relationship between Δ*θ* and Δ*n*.

In the incident beam with normal incidence (*θ* = 0°) and a corresponding transmission peak wavelength *λ*_C_ of 1550 nm, the refractive index of each dielectric layer of the porous silicon increased (Δ*n* = 0.01) when a biological response occurred in the PSM device. Thus, the transmission spectrum experienced a redshift, and the transmission peak wavelength of the transmitted light underwent a corresponding increase Δ*λ*_C_ of 11.7 nm. These results, labelled as cases 1 and 2, appear in Fig. 3(b). When the incident angle of the light source increased to 9.37°, the transmission peak *λ*_C_ of the PSM remained unchanged from the condition of vertical incidence, indicating that these two transmission peaks overlapped, as shown in Fig. 3(b) by cases 3 and 1, respectively. Figure 3(b) also shows that for the PSM device, when Δ*n* = 0.01, Δ*λ*_C_ = 11.7 nm. This approsch is the common method to obtain the refractive index change from the transmission spectrum. However, another new method is proposed for measuring the refractive index change of PSM devices. A single-wavelength infrared laser, with wavelength *λ*_i_ equal to the designed *λ*_C_, is used to irradiate the PSM device at normal incidence without reflection. When the PSM device with a laser vertically incident shows changes in its refractive index because of the biological reaction, the transmitted light will be weakened. If the incident laser beam is adjusted to a certain angle of *θ*, all of the light will be transmitted. Thus, the refractive index change Δ*n* caused by the change in the incident angle of the laser Δ*θ* can be obtained. Figure 4 shows, in theoretical calculations, the relationship between the refractive index Δ*n* and Δ*θ*.

The PSM refractive index increases by Δ*n* because of the antigen-antibody reaction. When the change in refractive index Δ*n* increases to 10^−4^, we can use the transfer matrix method to calculate that Δ*θ* = 0.046° and Δ*λ*_C_ = 0.05 nm. The resolution of the general angle measuring instrument is 1, that is, 0.0167°. It can detect a change of value Δ*n* of 10^−4^. The resolution of the optical experiment equipment for laboratory spectral detection (U-4100 UV-Vis-NIR Double Beam Spectrophotometer, Hitachi High-Technologies Corporation, Tokyo, Japan) is 0.1 nm; thus, it cannot detect a Δ*n* of 10^−4^. An expensive spectrophotometer with a resolution of 0.01 nm would be required to detect such an increase in Δ*n*.

**Figure 5:**
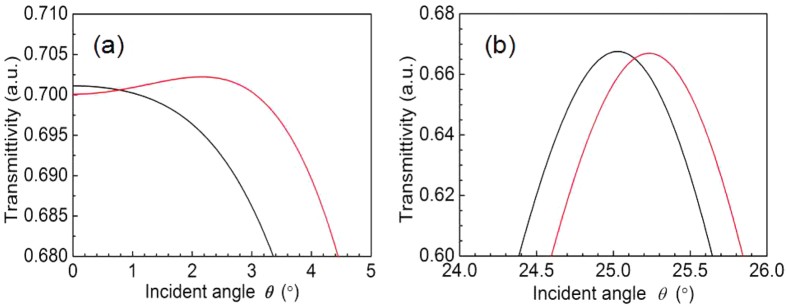
Transmission angular spectra (**a**) near 0° and (**b**) near 25°.

Figure 5 shows both the change in incident angle and the microcavity on both sides of the Bragg layers over six cycles of laser transmittivity. The black lines in the graph show the transmitted angular spectra, indicating that the refractive index of the porous silicon microcavity device remains unchanged. The red lines indicate the transmitted angular spectra of the porous silicon microcavity device after the refractive index changed, where Δ*n* = 0.001. In cases where the laser beam was incident near an angle of 25°, a detectable amount of movement for the transmitted angular spectrum of PSM was more obvious.

## Results and Discussion

**Figure 6:**
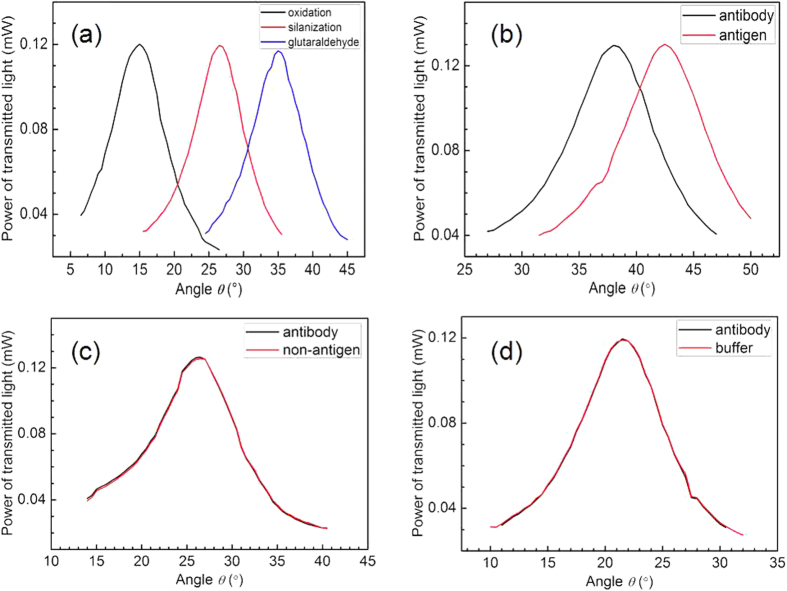
Power of transmitted light under various conditions. (**a**) Transmission angular spectra corresponding to changes in the PSM functional steps: after oxidation, after silanization, and after the addition of glutaraldehyde. (**b**) The PSM transmission angular spectra changes for the hydatid antigen. (**c**) Negligible PSM transmission angular spectra changes for a non-hydatid antigen. (**d**) No change in the PSM transmission angular spectra reaction to the buffer solution.

After each step in the function of the PSM, the oblique incident angle was used to detect and confirm each step of the chemical connection. Figure 6(a) shows the changes in angle with the PSM function as each step occurred. They are as follows, from left to right: after oxidation, after silanization, and after the addition of glutaraldehyde. Figure 6(b) illustrates the detection of the hydatid antibody in the PSM. The transmitted angular spectrum shifted approximately 4.54° after the PSM in which the probe was immobilized was exposed to 2 × 10^−6^ mg/ml of hydatid antigen. The effective refractive index change was due primarily to the effective combination of the hydatid antigen and immobilized probe in the PSM device. To demonstrate specificity, Fig. 6 (c) and (d) show the transmitted angular spectra exhibiting a negligible shift after exposure to a non-hydatid antigen and to a buffer solution (which must be flushed with DI water), respectively. The negligible shifts in the angle spectra may arise from the fact that the probe and non-hydatid antigen do not combine in the PSM, thus yielding no change in the effective refractive indices.

**Figure 7:**
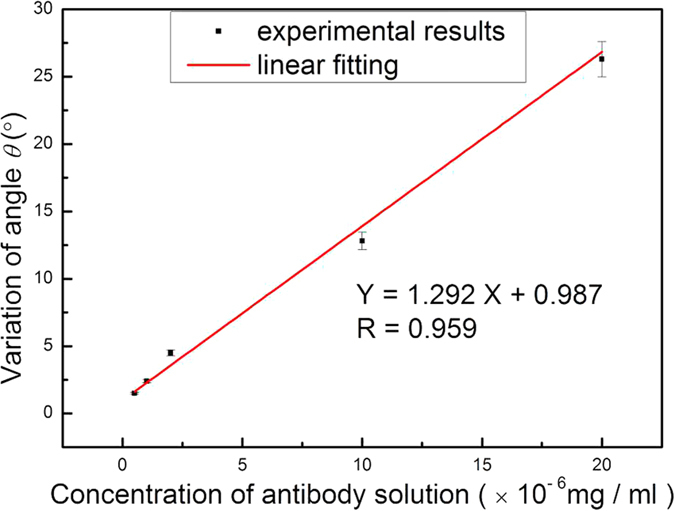
Linear relationship diagram for transmission angular spectrum changes with concentrations of hydatid antigen varying from 0.5 × 10^−6^ mg/ml to 2.0 × 10^−5^ mg/ml.

Figure 7 shows the shift in the transmission angular spectra for the hydatid antigen at different concentrations: 0.5 × 10^−6^, 1.0 × 10^−6^, 2.0 × 10^−6^, 1.0 × 10^−5^, and 2.0 × 10^−5^ mg/ml (Table 1). These results indicate that the angle variations corresponding to these concentrations were 1.52°, 2.46°, 4.54°, 12.88°, and 26.36°, respectively. Figure 7 also demonstrates a strong linearity (R = 0.959) between the shift of the transmitted angular spectrum and the concentration of hydatid antigen in the range from 0.5 × 10^−6^ mg/ml to 2 × 10^−5^ mg/ml. The equation of linear regression was determined to be Y = 1.292 X + 0.987, where Y represents the shift of the transmitted angular spectrum and X the concentration of hydatid antigen. Taking into account the stability of the light detector in this experiment and the interface roughness of each layer in the PSM, the actual angle measurement resolution was approximately 0.2°. Therefore, the detection limit of the biosensor was 0.16 ng/ml. Several relevant bioassays based on porous silicon have been reported: a nanoscale porous silicon microcavity biosensor for novel label-free tuberculosis antigen-antibody detection with a limit of 5.4 μg/ml^24^; an antifreeze protein detection using Rhodamine B as a photoluminescence label in porous silicon with a limit of 16.5 ng/ml^25^; and a novel multilayered porous silicon-based immunosensor for determining hydroxy-safflor yellow A with a limit of 0.78 ng/ml^26^. One study reports a porous silicon resonant microcavity biosensor for matrix metalloproteinase detection with a limit as low as 7.5 × 10^−19^ M^27^ because this biosensor is based on fluorescence detection, the limit of detection is lower.

**Figure 8:**
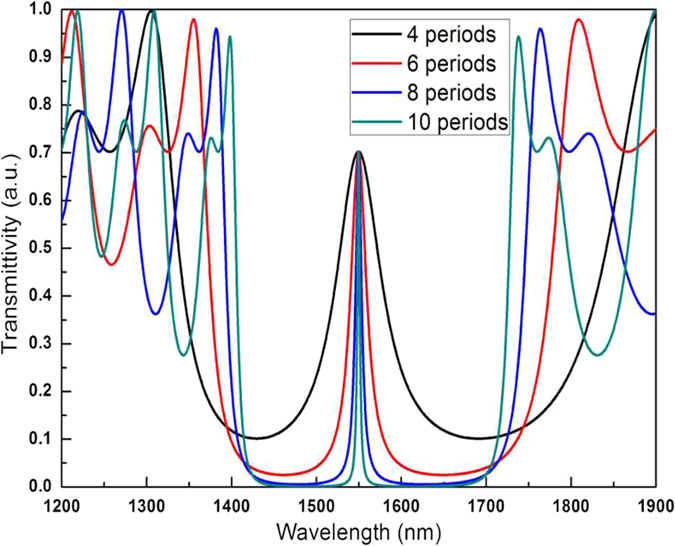
PSM transmission spectra of different periods.

The full width at half maximum (FWHM) of the transmitted angle spectrum will change with differences in the Bragg layer cycles and will affect the amount of change in the refractive index arising from changes caused by transmittance. If too few cycles of the Bragg layers occur on both sides of the microcavity, then the lower number of cycles will result in deterioration of the photonic crystal device characteristics, an increase in the FWHM of the transmitted angular spectrum, and a decrease in detection sensitivity. An excessive number of cycles makes it difficult for biomolecules to integrate into each layer of the PSM device. In the bioassay of hydatid disease, experiments indicated that the detection result was optimized if the Bragg layer was six cycles. Figure 8 shows the PSM transmission spectra of different periods. The FWHM of the transmission spectra becomes narrower with increasing cycles.

This paper proposed biosensors based on the PSM optical transmission angular spectra detection method to achieve the detection of the 43 kDa hydatid disease antigen. At a biosensor sensitivity of 1.30°/ng/ml, the detection limit was 0.16 ng/ml. The transmission angular spectra measurement method was adopted in this experiment, mainly to avoid the absorption of porous silicon and to increase the detection sensitivity. Thus, no light damage occurred in the biological sample. More importantly, the detection process was easier. Therefore, this optical biosensor, based on PSM and using the near-infrared transmitted angular spectrum for the detection of cystic hydatid disease, can offer a fast, convenient, and economical means of detection; thus, it can be widely promoted and applied in remote pastoral areas.

## Additional Information

**How to cite this article:** Li, P. *et al*. Hydatid detection using the near-infrared transmission angular spectra of porous silicon microcavity biosensors. *Sci. Rep.* **7**, 44798; doi: 10.1038/srep44798 (2017).

**Publisher's note:** Springer Nature remains neutral with regard to jurisdictional claims in published maps and institutional affiliations.

## Figures and Tables

**Table 1:** Experimental datas.

	Transmittance angle *θ* (°)						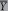 (°)	*S* (°)	*RSD* (%)	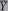 ± *S* (°)
	*Y*_1_	*Y*_2_	*Y*_3_	*Y*_4_	*Y*_5_	*Y*_6_				
Probe + antigen (0.5 × 10^−6^ mg/ml)	1.64	1.44	1.50	1.56	1.58	1.40	1.52	0.09	6.0	1.52 ± 0.09
Probe + antigen (1.0 × 10^−6^ mg/ml)	2.62	2.46	2.38	2.56	2.32	2.42	2.46	0.11	4.5	2.46 ± 0.11
Probe + antigen (2.0 × 10^−6^ mg/ml)	4.20	4.64	4.58	4.36	4.88	4.58	4.54	0.24	5.3	4.54 ± 0.24
Probe + antigen (1.0 × 10^−5^ mg/ml)	13.26	13.34	12.38	13.32	12.30	12.68	12.88	0.49	3.8	12.88 ± 0.49
Probe + antigen (2.0 × 10^−5^ mg/ml)	27.28	25.10	26.98	28.24	25.34	25.22	26.36	1.32	5.0	26.36 ± 1.32

The average value of the experiment as a result. Sensitivity and reproducibility of the PSM biosensor after exposure to different concentration of hydatid antigen in Fig. 7. *Y*_1_, *Y*_2_, *Y*_3_, *Y*_4_, *Y*_5_, *Y*_6_ are transmittance angle *θ* corresponding to these six samples, 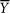 is average value of transmittance angle *θ*, *S* is standard deviations, and *RSD* is relative standard deviation, where 
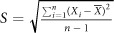
, 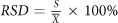.
